# Safety Behavior and Transition Shock among Newly Graduated Nurses: The Mediating Role of Feedback-Seeking Behavior

**DOI:** 10.1155/2023/9699240

**Published:** 2023-10-23

**Authors:** Yan Zhang, Xilin Yu, Xiaohong Lu, Yalin Tang, Wenbin Jiang, Qiaofeng Wei, Lili Wei

**Affiliations:** ^1^Department of Nursing, The Affiliated Hospital of Qingdao University, Qingdao, China; ^2^School of Nursing, The Hong Kong Polytechnic University, Hong Kong, China; ^3^School of Nursing, Qingdao University, Qingdao, China; ^4^Department of Cardiology, The Affiliated Hospital of Qingdao University, Qingdao, China; ^5^Department of Nursing and Hospital Infection Management, The Affiliated Hospital of Qingdao University, Qingdao, China; ^6^Department of Nursing, Laixi Municipal Hospital, Qingdao, China; ^7^Office of the Dean, The Affiliated Hospital of Qingdao University, Qingdao, China

## Abstract

**Aim:**

To determine the relationship between transition shock and safety behavior among newly graduated nurses (NGNs) and explore the mediating role of feedback-seeking behavior.

**Background:**

The safety behavior of NGNs plays a vital role in improving patient safety in clinical situations. The direct effect of the transition shock experienced by these nurses on safety behavior remains limited, and little is known about the mediating effect of feedback-seeking behavior.

**Methods:**

A descriptive correlational research design was conducted with a cross-sectional sample of nurses in China using an online survey. A convenience sample of 575 nurses from 17 hospitals completed the questionnaires. Correlation analysis and structural equation modeling were used to examine the hypotheses.

**Results:**

The sampled NGNs' safety behavior score was 55.35 ± 5.46. Their transition shock was negatively associated with safety behavior (*β* = −0.225, *p* < 0.001). In contrast, feedback-seeking behavior was positively related to safety behavior (*β* = 0.502, *p* < 0.001). The feedback-seeking behavior partially mediated the relationship between transition shock and safety behavior, and the mediating effect was 58.29%.

**Conclusions:**

The results emphasized that the relationship between NGNs' transition shock and safety behavior is mediated by feedback-seeking behavior. *Implications for Nursing Management*. Interventions focusing on transition shock relief could help improve NGNs' safety behavior. This study highlights the importance of encouraging feedback-seeking behavior to improve patient safety outcomes, especially for junior nurses. It can therefore be assumed that the nursing managers' training of NGNs in special skills, such as feedback-seeking, may be conducive to their positive coping and contribute to forming safety behaviors.

## 1. Introduction

Patient safety is a fundamental priority of global healthcare systems. However, delivery of safe care remains one of the greatest challenges in clinical practice [1].

Nursing-related adverse medical events accounted for approximately 40% of these incidents [2]. Therefore, practicing appropriate safety behavior is vital for nurses in ensuring that patients' safety goals are realized. Safety behavior refers to a series of actions conducted by nurses in their work to protect patients from harm or promote patient safety [3]. There remain obstacles to nurses' implementation of appropriate safety behavior such as poorly staffed hospitals, lack of appropriate nurse engagement, ineffective leadership, and burnout that can negatively impact nurses' safety behavior [4, 5]. Most studies to date have focused on the nursing population as a whole, and there has been less research conducted on specific groups [6, 7]. However, the times of new staff inductions accounted for more preventable errors than at other times of the year [2, 8].

Newly graduated nurses (NGNs) are defined as those with between 0 and 24 months of work/clinical experience according to the Royal College of Nursing (RCN) [9] or those within three years of graduation [10]. NGNs comprise an increasing proportion of nursing staff today, which may be a response to both concerns about critical shortages [11] and nurses' high turnover intentions [12]. The ability of NGNs to positively influence patient outcomes may be hampered by their unskilled techniques and limited clinical experience [13]. Furthermore, NGNs often show different intentions regarding the promotion of medication safety and attitudes towards medication management, which may be attributed to differences in education currently provided at the undergraduate level [14]. It is imperative that NGNs are equipped with the clinical competence and experience necessary to prevent poor patient outcomes. For nursing managers, understanding the influencing mechanism of NGNs' safety behavior is essential. This is also the basis to explore new paths to improve their safety behavior and promote the intervention development aimed at reducing the incidence of adverse events.

NGN transition has been at the forefront of nursing research in recent years, as the competence of NGNs has been questioned, along with the observed latent relationship between NGNs and a high rate of adverse patient events. According to Duchscher's transition shock model, transition shock emerges when nurses move from the known role of students to the less familiar role of professional nurses [15]. For NGNs, the transition to practice is a highly turbulent time that brings continued challenges. During this time, they are vulnerable newcomers who require understanding and support from their more experienced colleagues [16]. The sudden responsibility imposed on NGNs as registered nurses can make them feel overwhelmed, stressed, and diminished in confidence [17, 18]. To address some of these difficulties experienced, the transition shock model proposes that enhancing NGN's transition to practice through targeted education can help to improve patient safety [19].

Feedback-seeking behavior is defined as the conscious devotion of effort towards determining the correctness and adequacy of one's behavior to attain valued goals [20]. Employees could become proactive by seeking feedback in the socialization process, adopting norms, and manifesting appropriate behavior accepted in the organization [21]. Research on transition shock mainly focused on employees, medical students, and interns [21–23]. However, the evidence of new nurses' feedback-seeking behavior is limited. Thus, it is necessary to explore the feedback-seeking behavior of new nurses, which may provide a new perspective for nursing management.

In recent studies, nurses' transition shock has been negatively linked to feedback-seeking behavior [22]. Previous studies have reported that feedback has a synergistic influence on safety-related behavior changes and can be used as a cogent tool in safety incentive systems [23]. Feedback was also suggested as an important factor in supporting positive transition for NGNs [24]. A qualitative study reported that seeking feedback could help NGNs obtain positive experiences and facilitate a successful transition [16]. Prior studies focused less on new nurses. For the mediating effect of feedback-seeking behavior on the relationship between transition shock and patient safety, research remains scarce. According to the COM-B (Capability, Opportunity, Motivation, Behavior) framework developed by Michie [25], to perform a specific behavior, individuals must be physically and psychologically capable (C) of possessing adequate skills and knowledge, have opportunities (O) that enable or prompt the behavior, and also have reflective or automatic motivation (M) . In this study, we hypothesized that except for motivation (M), transition shock (C) and feedback-seeking (O) would both play important roles in the endorsement of safety behavior (B). Hence, we hypothesized that (1) NGNs' transition shock is negatively related to safety behavior; (2) feedback-seeking behavior is positively linked to safety behavior; and (3) feedback-seeking behavior serves to mediate the association between transition shock and safety behavior.

## 2. Methods

### 2.1. Design

A multicenter cross-sectional research study was conducted.

### 2.2. Sample

The NGNs that participated in the study were selected by convenience sampling on October 10, 2022. The inclusion criteria were as follows: (a) being a full-time registered nurse, (b) holding either a permanent or contracted role, (c) being formally employed in either a private or public clinical unit, (d) having no more than three years of work experience, and (e) being willing to participate. Nurses who were absent from work because of illness or personal leave were excluded.

The minimum sample size was calculated using the PASS program (version 11.0), which indicated that a sample size of 154 achieved 90% power to detect an R-Squared value of 0.10, which was attributed to five independent variables using an F-test with a significance level (alpha) of 0.05 [26]. Considering the nonresponses of the target sample and missing data, the calculated sample size was 200. However, it was difficult to estimate the total number of NGNs who received the survey link, as the link was disseminated by administrators working in the nursing departments of each hospital. The final sample size was 600. A total of 575 valid questionnaires were used for data analysis, with an effective response rate of 95.83%.

### 2.3. Procedure

The online survey was conducted using Wenjuanxing (http://www.wjx.cn), the largest domestic online survey platform in China. The researchers compiled a standardized set of instructions, including information on the purpose and significance of the study. Electronic questionnaires with formatted instructions were sent using WeChat groups to participants who met the inclusion criteria. All survey items were answered before submission to ensure the effectiveness of the data collection. A total of 600 NGNs in 17 hospitals participated in this study.

### 2.4. Instruments

#### 2.4.1. General Demographic Questionnaire

The participants' demographic data were collected using a general demographic questionnaire, which included gender, age, ethnicity, employment type, educational level, marital status, prior working experience, whether they were parents, whether their parents were medical workers, the average number of night shifts worked per month, whether they were independent on duty, and their level of participation in systematic patient safety training.

#### 2.4.2. Nurse Safety Behavior Questionnaire

The Nurse Safety Behavior Questionnaire (NSBQ) was used to calculate the NGNs' safety behavior level. Shih designed the questionnaire [3] and Rong translated it into Chinese [27]. This questionnaire has been widely used to investigate nurses' work performance in relation to avoiding patient harm and improving patient safety. The NSBQ consists of 12 items in total, and a 5-point Likert scale is used to calculate the total score. Responses for this study were measured on a scale ranging from 1 (never) to 5 (always). Higher scores indicated better nurses' performance regarding patient safety behavior. The revised Chinese version of the NSBQ questionnaire has shown good reliability and validity, with a Cronbach's *α* of 0.931 in newly recruited nurses [27]. In this study, Cronbach's *α* was 0.894.

#### 2.4.3. Feedback-Seeking Behavior Scale

The Feedback-Seeking Behavior Scale (FSBS) was developed by Callister et al. [28]. The FSBS is an 11-item scale divided into four dimensions of feedback-seeking behavior: leader inquiry (two items), leader observation (two items), colleague observation (three items), and colleague inquiry (four items). For this study, the items were evaluated on a 7-point scale ranging from 1 (completely inconsistent) to 7 (completely consistent), with higher scores indicating better feedback-seeking behavior. The FSBS was previously translated into Chinese and used for a survey on the feedback-seeking behavior of employees in Chinese enterprises, and its Cronbach's *α* was 0.890 [29]. Cronbach's *α* of the scale in this study was 0.961, and was respectively, 0.940, 0.896, 0.954, and 0.940 for each dimension.

#### 2.4.4. Transition Shock of Newly Graduated Nurses Scale

Xue developed the Transition Shock of Newly Graduated Nurses Scale (TSNGNS) based on Duchscher's transition shock theory [30]. This scale consists of 27 items and is scored on a 5-point Likert scale (ranging from 1 = totally disagree to 5 = totally agree). This scale is grouped into four subscales: physical (six items), emotional (eight items), knowledge and skills (five items), and sociocultural and developmental (eight items). Higher scores indicate a stronger transition shock. Content validity was tested, and the item content validity indices all exceeded 0.86. The reported Cronbach's *α* coefficients varied from 0.86 to 0.94 [30]. Cronbach's *α* of the scale was 0.974, and was respectively, 0.929, 0.931, 0.910, and 0.947 for each dimension in the present study.

### 2.5. Statistical Analysis

Data were analyzed using IBM SPSS Statistics (version 26) and IBM SPSS AMOS (version 26). Categorical data, such as demographic data, were presented as frequencies and percentages. Continuous variables with normal distribution were expressed as mean ± standard deviations (M ± SD). An independent sample *t*-test and one-way ANOVA were used to compare the differences in safety behavior, transition shock, and feedback-seeking behavior according to each of the recorded demographic characteristics. Post hoc comparisons were performed for statistically significant associations using the Bonferroni test. Pearson's correlation analysis was used to determine the correlationsamong the three major variables. Regression analysis were conducted to estimate the direct effect of transition shock on nurses' safety behavior. Structural equation modeling with the advantage of dealing with relationships between multiple variables and distinguishing indirect effects from direct effects was chosen to examine the indirect effect of feedback-seeking behavior among NGNs.

### 2.6. Ethical Considerations

The Ethics Review Committee of the Affiliated Hospital of Qingdao University approved this study (Approval No. QDFYWZLL27973). After receiving approval from each of the hospitals involved in this study, online questionnaires were sent to the study participants along with informed consent forms. Their agreement to participate was asserted by choosing the “I agree” option ahead of filling in the questionnaires, which ensured that all respondents fully agreed to participate in this survey. Participants were assured that the questionnaires were collected anonymously, and that all individual information was strictly confidential. All methods were performed in accordance with the Strengthening the Reporting of Observational Studies in Epidemiology (STROBE) statement.

## 3. Results

### 3.1. Characteristics of Participants

Table 1 summarizes respondents' recorded characteristics. A total of 575 nurses, 461 females (80.2%) and 114 males (19.8%), participated in this study. The mean age of the participants was 23.94 ± 2.00 years. Participants were predominantly unmarried (89.0%) and had no children (95.5%). A total of 272 (47.3%) of the nurses surveyed had a bachelor's degree, 75% had worked for <2 years, and most (81.2%) were independent on duty. The proportion of temporary nurses was eight times more than that of permanent employment nurses (88.9% vs. 11.1%). Most respondents (80.5%) had participated in systematic patient safety training.

### 3.2. Score of NGNs' Safety Behavior, Transition Shock, and Feedback-Seeking Behavior

Table 2 shows the descriptive statistics of the variables and the normality test results. The normality test results for the latent variable factors were verified using skewness and kurtosis. According to the standard proposed by Klein [31], the data recorded meet the criteria for an approximate normal distribution. The absolute values of the skewness coefficient and kurtosis coefficient were three and within eight, respectively.

The mean score of NGNs' safety behavior was 55.35 ± 5.46. The mean score of the transition shock scale was  3.01± 1.01, with the physical aspect scoring highest and social culture and development lowest. For the feedback-seeking behavior, the average score was 62.70 ± 12.60. The order of the four dimensions according to their score from high to low was as follows: colleague observation, leader observation, colleague inquiry, and leader inquiry.

### 3.3. Comparison of NGNs' Safety Behavior, Transition Shock, and Feedback-Seeking Behavior with Different Demographic Characteristics

The results of the *t*-test and ANOVA showed that employment type, educational level, whether the respondents have child or not, hospital working experience, average number of night shifts per month, and participation in systematic patient safety training significantly impacted NGNs' safety behavior. In the post hoc comparison, junior NGNs showed the highest level of safety behavior compared to bachelors (mean difference: 1.490; *p* = 0.003; Cohen's effect *d* = 0.28) and master's degree or above (mean difference: 5.011; *p* < 0.001; Cohen's effect *d* = 0.84). NGNs who worked for 25–36 months showed lower safety behavior compared to those who worked for 13–24 months (mean difference: 1.605; *p* = 0.043; Cohen's effect *d* = 0.31). Those who had 5 night shifts per month showed lower safety behavior compared to those with 3-4 night shifts per month (mean difference: 1.755; *p* = 0.009; Cohen's effect *d* = 0.34).

Employment type, educational level, average number of night shifts per month, and participation in systematic patient safety training significantly influenced the transition shock experienced by NGNs (*p* < 0.05). NGNs with a bachelor's degree showed higher transition shock compared to juniors (mean difference: 4.862; *p* = 0.014; Cohen's effect *d* = 0.24). Those with ≥ 5 night shifts per month showed higher transition shock compared to their counterparts without night shift (mean difference: 6.764; *p* = 0.023; Cohen's effect *d* = 0.42).

Furthermore, educational level, parental status, average number of night shifts per month, and participation in systematic patient safety training also had a meaningful impact on NGNs' feedback-seeking behavior. NGNs with bachelor's degree showed higher feedback-seeking behavior compared to juniors (mean difference: 3.933; *p* = 0.001; Cohen's effect *d* = 0.32). Those who worked ≥5 night shifts per month showed lower feedback-seeking behavior compared to those with 3-4 night shifts per month (mean difference: 4.240; *p* = 0.005; Cohen's effect *d* = 0.34). The details are presented in Table 1.

### 3.4. Correlations between Variables

The correlations between the latent variables were analyzed using Pearson's correlation coefficient analysis (Table 2). The transition shock experienced by NGNs was negatively and significantly related to their feedback-seeking behavior (*r* = −0.195, *p* < 0.01) and safety behavior (*r* = −0.223, *p* < 0.01). Additionally, NGNs' feedback-seeking behavior had a positive relationship with their safety behavior (*r* = 0.502, *p* < 0.01).

### 3.5. Results of the Structural Equation Model

First, a confirmatory factor analysis was conducted to identify how well the observed variables represented the latent variables in this study. The standardization factors of all constructs were shown to be higher than the recommended value of 0.7 [31] and the *t* value was at least 1.96, indicating that the construct validity of the scales was supported. The Average Variance Extracted (AVE) values were >0.5, and construct reliability CR values were >0.7, which indicated that the measurement tool had good convergent validity [32] (Table 3).

The fitness analysis of the theoretical model showed that it proved a satisfactory fit (CMIN/DF(*χ*^2^/d*f*) = 2.275 < 3, GFI = 0.982 > 0.9, AGFI = 0.961 > 0.9, RSMSEA = 0.047 < 0.05, IFI = 0.993 > 0.9, NNFI = 0.942 > 0.9, CFI = 0.993 > 0.9).

**Figure 1:**
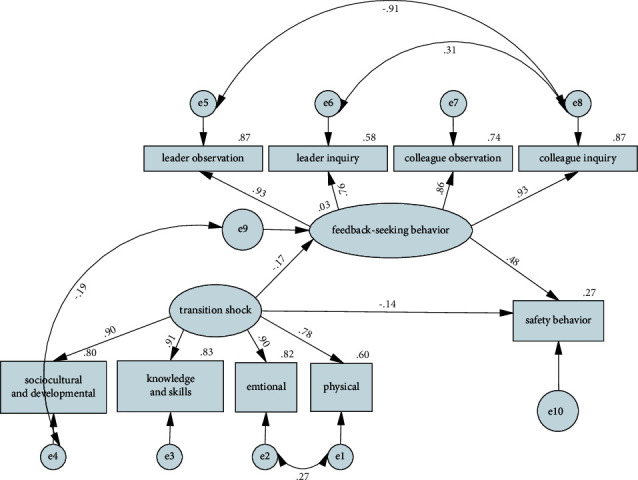
Path diagram of the NGNS' safety behavior model (standardized regression coefficients).

The standardization path coefficient and *t* values of the structural equation model were verified to determine whether there were direct or indirect relationships between the variables (Table 4).  Figure 1 shows the pathways between the variables in the nurses' safety behavior model. The NGNs' transition shock was negatively associated with feedback-seeking behavior (*β* = −0.170, *p* < 0.001). Their feedback-seeking behavior was positively associated with safety behavior (*β* = 0.447, *p* < 0.001). Meanwhile, the NGNs' transition shock had a direct effect on the nurse safety behavior model (*β* = −0.140, *p* < 0.001). In the bootstrapping test, the 2,000 bootstrapping resamples revealed that the 95% CI recorded for the indirect effect of transition shock on safety behavior through feedback-seeking behavior did not include zero (−0.808/−0.274), indicating that the indirect effect was significant. Therefore, these findings indicate that NGNs' feedback-seeking behavior acted as a mediator in the relationship between their transition shock and safety behaviors.

## 4. Discussion

This study investigated the influence of transition shock on NGNs' safety behavior and determined the mediating role of feedback-seeking behavior between safety behavior and transition shock.

The NGNs who participated in this study exhibited a level of safety behavior consistent with previous research conducted in this field [33]. As shown in the results, the average score of NGNs' safety behavior was 55.35 ± 5.46, which was also similar to that of 454 newly recruited nurses in Shandong Province with <2 years of work experience (56.56 ± 5.19) [33]. The reasons may be the high similarity of participants; both studies involved NGNs who had worked for less than two or three years. NGNs' work experience was significantly related to their safety behavior, which concurred with that of Chu et al. [34]. The longer the employment duration, the better the safety behavior recorded (*t* = 3.560, *p* = 0.015) [34]. Adverse events may occur because of the incompetency of individual healthcare professionals with limited experience in ensuring patient safety, inadequate skills, or knowledge, or because of untargeted training programs, resulting in poor safety behavior [34–36]. Compared with senior nurses with rich experience and long working time, the safety behavior of NGNs needs to be improved. An implication of this finding is the possibility that attending the systematic patient safety behavior training may improve NGNs' professional competence and reduce the incidence of adverse events.

The transition to practice is a key learning period that sets new nurses on the path to becoming expert practitioners [10]. During this process, NGNs face immense physical and psychological shock owing to their unfamiliarity with clinical work [37]. The one-way ANOVA results showed that the average number of night shifts per month was one of the main factors that influenced the transition shock levels of NGNs. This finding is consistent with the results obtained by Zhang et al. [38]. Night-shift work is related to sleep deprivation and physical and psychological burnout, which could in turn aggravate the transition impact [38, 39]. Night-shift work is also associated with an increased prevalence of mood disorders [40]. Furthermore, labile emotions were constantly expressed by NGNs during the initial transition stage [15] and were found to be closely related to nurses' transition shock [39, 41]. This raises the possibility that delaying of the start to night shift or reducing its frequency may allow NGNs to adapt to the new working environment and reduce the degree of physical and psychological burnout. Meanwhile, nursing managers need to pay attention to NGNs' emotional status and recommend necessary psychological counseling to reduce the accumulation of their negative emotions. Of a possible mean value of five, our results also showed that newly graduated nurses experienced moderate transition shock (3.01 ± 1.01). The higher the NGNs' educational level, the higher the transition shock. Analysis showed that the higher the nurses' educational levels, the higher their individual requirements and the stronger their sense of achievement. The mismatch between the expectations of professional practice and the reality experienced leads to a lack of confidence in the ability to work independently and the inability to continuously deal with many tasks simultaneously [37–39]. This indicates that nursing management needs to focus on NGNs with higher educational levels , and pay attention to theirself-regulating and environmental support. Participating in systematic patient safety training was another important factor that influenced not only NGNs' level of transition shock but also affected their feedback-seeking and safety behavior in this study. Having less work experience may cause a lack of safety consciousness among NGNs [42]. Thus, safety training and efforts to increase professional knowledge may be undertaken to improve NGNs' comprehensive abilities.

The transition shock experienced by NGNs directly and negatively affected feedback-seeking behavior [22, 43]. A reasonable explanation may be that junior nurses (nurses with <5 years of clinical nursing experience) have worked for longer than NGNs, meaning they are more familiar with the work itself, have less stress, and tend to choose positive and active coping styles. Hence, there is a need for nursing managers to encourage new nurses to adopt more feedback-seeking behavior through relevant training and the cultivation of a feedback-exchange culture in hospitals. Previous studies also have confirmed that reducing transition shock enables young nurses to find a better balance in their work life and achieve better physical, psychological, and social health [44, 45], which could in turn enhance their initiative to seek feedback from the workplace. Previous findings from qualitative content analysis have reported the importance of regular feedback conversations in improving NGNs' correct clinical behavior [16]. Therefore, we infer that supportive feedback-seeking cultures may be maintained by enhancing transition facilitation programs for NGNs.

NGNs have previously identified deficiencies in the work environment as a major source of frustration during this transition [46]. Their transition shock was a direct, significant predictor of safety behavior, indicating that NGNs who experienced less reality shock in the transition phase were more competent in avoiding patient harm and improving patient safety. This study's results also support those of Hampton, who argued that the transition shock experienced by NGNs has a significant influence on missed nursing care, adverse events, and perceived quality of care [47]. Novice nurses who were not prepared for the inconsistencies that occur between academic training and professional nursing practices experienced significant internal conflict [48]. This mismatch has long been linked to negative nursing and patient outcomes [49]. To increase NGNs' ability to cope with the shock of transition, medical institutions may be required to accommodate an evolving program of mentorship, utilizing qualified nurse preceptors and experienced nurses.

Previous research has shown that feedback provision can ensure nurses' adherence to patient-safety principles [50]. This finding broadly supports the work of other studies in this area linking NGNs' feedback-seeking with their safety behavior at work. A systematic review summarized that nurses actively seeking regular practical feedback processes, interaction opportunities, and observation of peers and senior colleagues could improve their adherence to patient safety principles [51]. It is therefore likely that encouraging newcomers' feedback-seeking through special orientation programs, social events, and mentoring could reduce the probability of nursing error events for NGNs and enhance the effectiveness of interventions for safety and care quality. Improving nurses' patient safety behavior is an essential component of quality of care [52]. Nurses' positive safety behavior is strongly associated with a reduction in the key patient outcomes of falls, medication errors, pressure injuries, and healthcare-associated infections [53]. In line with our hypotheses, we found that feedback-seeking behavior acted as a partial mediator in the relationship between NGNs' transition shock and safety behavior. Previous studies focused more on a single variable, such as the impact of transition shock or feedback-seeking on nurses' safety behavior. This study is the first to reveal that reducing NGNs' transition shock and ensuring their smoother integration into the nursing workforce may increase the level of feedback-seeking behavior among nurses, which in turn motivates NGNs to engage in more safety behavior in practice. These provide further support for the hypotheses that pre-job orientation programs, through including knowledge about professional role transition, proposing preceptorship and peer support initiatives, and providing individualized transitional psychological counseling and feedback-seeking skills for NGNs, may benefit their safety behavior. These measures could establish a harmonious workplace culture and climate for NGNs and ultimately decrease the level of emotional, physical, sociocultural, developmental, and intellectual shock they experience.

### 4.1. Limitations

This study has several limitations. First, it adopted a cross-sectional research design, which could not provide cause-and-effect explanations. Thus, longitudinal studies examining NGNs' transition shock, feedback-seeking behavior, and safety behavior are recommended. Second, data were collected from 17 hospitals in China. This location and sample range may be regarded as too geographically limited for broad generalization. A further study with more focus on improving the generalization of the sample population and locations is therefore suggested. Third, we used convenience sampling and adopted self-reported measurements, which may have increased social desirability bias. Finally, this study investigated only feedback-seeking behavior as a mediator between NGNs' transition shock and safety behavior. Other mediators such as organizational support, safety knowledge and motivation, psychological capital, and perception of patient safety culture should be explored in future studies.

## 5. Conclusions

This study provides additional support for the hypotheses that NGNs' transition shock is negatively associated with safety behavior, and that feedback-seeking behavior is positively associated with NGNs' safety behavior. Transition shock indirectly affects NGNs' safety behavior through the mediating effect of feedback-seeking behavior. Management of NGNs' transition shock is essential to enhancing NGNs' feedback-seeking behavior, which ultimately promotes their safety behavior in clinical practice.

## 6. Implications for Nursing Management

Transition shock and feedback-seeking behavior are two vital variables that influence NGNs' safety behavior in practice. Our findings indicate that a successful transition from student to clinical nursing roles would make NGNs feel a less oppressive hierarchy among nursing staff and enhance their willingness to seek constructive feedback, which could in turn improve their overall safety behavior. This provides a new clue for nursing managers to improve the safety behavior of new nurses. For nursing managers, it is also important to take cluster measures to reduce the transition shock of new nurses. Futhermore, the corresponding measures to improve their feedback-seeking behavior not only help reduce the level of transition shock but also help new nurses to improve safety behavior. Therefore, compared to reducing their transition shock alone, this may significantly improve the safety behavior of new nurses.

## Figures and Tables

**Table 1:** Participants' demographics and differences in study variables (*N* = 575).

Characteristics	Category	No. (%)	Safety behavior			Transition shock			Feedback-seeking behavior		
			M ± SD	*t*/*F*	*p*	M ± SD	*t*/*F*	*p*	M ± SD	*t*/*F*	*p*
Gender	Female	461 (80.2)	55.20 ± 5.37	−1.38^a^	0.168	59.32 ± 19.21	−0.89^a^	0.373	62.52 ± 12.56	−0.67^a^	0.506
	Male	114 (19.8)	55.98 ± 5.80			61.21 ± 24.05			63.40 ± 12.82		

Ethnicity	Han	549 (95.5)	55.36 ± 5.48	0.15^a^	0.879	59.32 ± 20.30	−2.01^a^	0.045^*∗*^	62.76 ± 12.70	0.58^a^	0.565
	Other	26 (4.5)	55.19 ± 5.18			67.46 ± 18.00			61.30 ± 10.48		

Only child	Yes	161 (28.0)	55.43 ± 5.34	−0.23^a^	0.819	59.21 ± 21.35	0.35^a^	0.723	63.80 ± 12.33	−1.31^a^	0.191
	No	414 (72.0)	55.32 ± 5.51			59.88 ± 19.83			62.27 ± 12.70		

Employment types	Temporary	511 (88.9)	55.58 ± 5.41	2.90^a^	0.004^*∗∗*^	58.81 ± 20.10	−2.97^a^	0.003^*∗∗*^	62.97 ± 12.65	1.48^a^	0.139
	Permanent	64 (11.1)	53.50 ± 5.56			66.72 ± 20.29			60.50 ± 12.10		

Educational level	Junior college	284 (49.4)	56.22 ± 4.48	11.21^b^	<0.001^*∗∗*^	57.18 ± 21.32	4.41^b^	0.013^*∗*^	64.69 ± 12.21	7.13^b^	0.001^*∗∗*^
	Bachelor	272 (47.3)	54.73 ± 6.03			62.04 ± 19.07			60.75 ± 12.73		
	Master or above	19 (3.3)	51.21 ± 7.18			63.55 ± 16.05			60.84 ± 12.67		

Marital status	Married	63 (11.0)	55.94 ± 4.35	−0.90^a^	0.368	62.02 ± 21.30	−0.97^a^	0.334	64.51 ± 10.66	−1.21^a^	0.228
	Unmarried	512 (89.0)	55.28 ± 5.58			59.41 ± 20.12			62.48 ± 12.81		

Have child	None	549 (95.5)	55.28 ± 5.54	−2.23^a^	0.029^*∗*^	59.38 ± 20.02	−1.73^a^	0.084	62.47 ± 12.68	−2.52^a^	0.017^*∗*^
	Have	26 (4.5)	56.85 ± 3.27			66.41 ± 24.16			67.54 ± 9.86		

Working experience (months)	≤6	48 (8.3)	54.60 ± 6.18	2.77^b^	0.041^*∗*^	61.90 ± 22.24	1.57^b^	0.195	62.75 ± 11.72	0.96^b^	0.409
	7–12	200 (34.8)	55.38 ± 5.49			57.17 ± 19.35			62.78 ± 12.64		
	13–24	183 (31.8)	56.38 ± 4.37			59.90 ± 22.23			64.01 ± 12.86		
	25–36	144 (25.0)	54.77 ± 5.87			61.32 ± 18.95			61.67 ± 12.58		

Parents are medical workers	Yes	19 (3.3)	57.32 ± 4.22	−1.60^a^	0.111	49.71 ± 20.34	2.19^a^	0.029^*∗*^	66.74 ± 13.12	−1.42^a^	0.156
	No	556 (96.7)	55.28 ± 5.49			60.03 ± 20.18			62.56 ± 12.57		

Night shifts (per month)	0	102 (17.7)	55.45 ± 5.78	3.41^b^	0.017^*∗*^	54.82 ± 12.45	3.41^b^	0.017^*∗*^	63.56 ± 12.18	3.93^b^	0.009^*∗∗*^
	1-2	46 (8.0)	55.28 ± 5.00			63.04 ± 20.84			62.74 ± 12.02		
	3-4	150 (26.1)	56.47 ± 4.54			58.48 ± 22.36			65.24 ± 11.56		
	≥5	277 (48.2)	54.72 ± 5.78			61.59 ± 19.31			61.00 ± 13.18		

Independent on duty	Yes	467 (81.2)	55.39 ± 5.14	0.33^a^	0.741	60.18 ± 20.37	1.21^a^	0.226	62.71 ± 12.65	0.04^a^	0.970
	No	108 (18.8)	55.19 ± 6.68			57.56 ± 19.68			62.66 ± 2.45		

Systematic safety training	Yes	463 (80.5)	56.25 ± 4.41	6.30^a^	<0.001^*∗∗*^	58.55 ± 20.76	−3.10^a^	0.002^*∗∗*^	64.39 ± 11.55	5.98^a^	<0.001^*∗∗*^
	No	112 (19.5)	51.63 ± 7.46			64.43 ± 17.32			55.70 ± 14.31		

On an 8-hour basis, the 12 h night shift refers to 1.5 night shifts. ^*∗*^*p* < 0.05; ^*∗∗*^*p* < 0.01. ^a^t-test for the independent group; ^b^one-way ANOVA.

**Table 2:** Scores of safety behavior, transition shock, and feedback-seeking behavior and correlations (*N* = 575).

Latent variable	Observed variable	M ± SD	Min/max	Feedback-seeking behavior	Safety behavior
Safety behavior		55.35 ± 5.46	11.00/77.00		1

Transition shock		3.01 ± 1.01	1.00/5.00	−0.195^*∗∗*^	−0.223^*∗∗*^
	Physical	3.39 ± 1.13	1.00/5.00		
	Emotional	3.02 ± 1.07	1.00/5.00		
	Knowledge and skills	2.99 ± 1.09	1.00/5.00		
	Sociocultural and developmental	2.64 ± 1.14	1.00/5.00		

Feedback-seeking behavior		62.70 ± 12.60	11.00/77.00	1	0.502^*∗∗*^
	Leader observation	5.86 ± 1.10	1.00/7.00		
	Leader inquiry	5.28 ± 1.64	1.00/7.00		
	Colleague observation	5.94 ± 1.07	1.00/7.00		
	Colleague inquiry	5.65 ± 1.31	1.00/7.00		

^*∗∗*^ *p* < 0.001.

**Table 3:** Confirmatory factor analysis of the measurement model.

Latent variable	Observed variable	*B*	*β*	SE	*t* value	AVE	CR
NGNs' transition shock						0.76	0.93
⟶	Physical	1.00	0.78				
	Emotional	1.11	0.91	0.04	27.73^*∗∗*^		
	Knowledge and skills	1.13	0.91	0.05	23.76^*∗∗*^		
	Sociocultural and developmental	1.16	0.90	0.05	23.41^*∗∗*^		

Feedback-seeking behavior						0.76	0.93
⟶	Leader observation	1.00	0.93				
	Leader inquiry	1.22	0.76	0.05	22.59^*∗∗*^		
	Colleague observation	0.90	0.86	0.03	27.36^*∗∗*^		
	Colleague inquiry	1.19	0.93	0.04	27.50^*∗∗*^		

^*∗∗*^ *p* < 0.001.

**Table 4:** Path analysis between variables of the study model.

Path	*β*	*SE*	*t value*	*p*	95% CI
Transition shock to feedback-seeking behavior	−0.170	0.051	−3.868	<0.001	−0.305/−0.098
Feedback-seeking behavior to safety behavior	0.477	0.205	12.37	<0.001	1.935/3.332
Direct effect of transition shock on safety behavior	−0.140	0.236	−3.693	<0.001	−1.437/−0.238
Indirect effect of transition shock on safety behavior	−0.502			<0.001	−0.808/−0.274
Total effect	−1.373			<0.001	−0.808/−0.275

## Data Availability

The data used to support the findings of this study are available from the corresponding authors upon reasonable request.

## References

[1] World Health Organization (2018). 10 Facts on Patient Safety. http://www.who.int/features/factfiles/patient_safety/en/.

[2] Chen J., Zhang D. W., Jin X. (2018). Characterization of the a gene and its role in the NF-*κ*B signaling pathway of s. *Frontiers in Physiology*.

[3] Shih C. P., Chang L. Y., Chen J. C., Ng C. J., Reinfeld W., Hsu K. H. (2008). The factors influencing safety behavior of medical staffs in emergency room of a medical center in Taiwan. *Journal of Management and Business Research*.

[4] Prapanjaroensin A., Patrician P. A., Vance D. E. (2017). Conservation of resources theory in nurse burnout and patient safety. *Journal of Advanced Nursing*.

[5] El-Gazar H. E., Zoromba M. A., Zakaria A. M., Abualruz H., Abousoliman A. D. (2022). Effect of humble leadership on proactive work behaviour: the mediating role of psychological empowerment among nurses. *Journal of Nursing Management*.

[6] Wang L., Lu H., Dong X. (2020). The effect of nurse staffing on patient-safety outcomes: a cross-sectional survey. *Journal of Nursing Management*.

[7] Jun J., Ojemeni M. M., Kalamani R., Tong J., Crecelius M. L. (2021). Relationship between nurse burnout, patient and organizational outcomes: systematic review. *International Journal of Nursing Studies*.

[8] Duckett S., Moran G. (2018). Why You Should Avoid Hospitals in January. https://grattan.edu.au/news/why-you-should-avoid-hospitals-in-january/.

[9] Buchan J. (2007). Nurse Workforce Planning in the UK, A Report for the Royal College of Nursing. https://www.researchgate.net/profile/James-Buchan/publication/256288558_Workforce/links/a85e530a8de92f626000000/Workforce.pdf.

[10] Murray M., Sundin D., Cope V. (2018). New graduate registered nurses’ knowledge of patient safety and practice: a literature review. *Journal of Clinical Nursing*.

[11] Haddad L. M., Annamaraju P., Toney-Butler T. J. (2023). *Nursing Shortage*.

[12] Magbity J. B., Ofei A. M. A., Wilson D. (2020). Leadership styles of nurse managers and turnover intention. *Hospital Topics*.

[13] Al-Rawajfah O. M., AlBashayreh A., Sabei S. D. A., Al-Maqbali M., Yahyaei A. A. (2023). Role transition from education to practice and its impact on the career futures of Omani nurses. *Nurse Education in Practice*.

[14] Fernandez R., tenHam-Baloyi W., Mathew E. (2023). Predicting behavioural intentions towards medication safety among student and new graduate nurses across four countries. *Journal of Clinical Nursing*.

[15] Duchscher J. E. (2009). Transition shock: the initial stage of role adaptation for newly graduated registered nurses. *Journal of Advanced Nursing*.

[16] Regan S., Wong C., Laschinger H. K. (2017). Starting Out: qualitative perspectives of new graduate nurses and nurse leaders on transition to practice. *Journal of Nursing Management*.

[17] Murray M., Sundin D., Cope V. (2020). A mixed-methods study on patient safety insights of new graduate registered nurses. *Journal of Nursing Care Quality*.

[18] Kaldal M. H., Conroy T., Feo R., Grønkjaer M., Voldbjerg S. L. (2022). Umbrella review: newly graduated nurses’ experiences of providing direct care in hospital settings. *Journal of Advanced Nursing*.

[19] Murray M., Sundin D., Cope V. (2019). Benner’s model and Duchscher’s theory: providing the framework for understanding new graduate nurses’ transition to practice. *Nurse Education in Practice*.

[20] Ashford S. J. (1986). Feedback-seeking in individual adaptation: a resource perspective. *Academy of Management Journal*.

[21] Bălăceanu A., Vîrgă D., Maricuțoiu L. (2021). Feedback-seeking behavior in organizations: a meta-analysis and systematical review of longitudinal studies. *Spanish Journal of Psychology*.

[22] Ma Z. Y., Chen F., Lu N. N., Li X. L., Zhang C. (2022). Research on relationship among transition shock, adversity quotient and feedback seeking behavior of newly graduated nurses. *Chinese Journal of Nursing*.

[23] Lian Y., Zhang C. M. (2020). Correlation between nursing interns’ transformation impact and feedback seeking behavior. *Journal of Nursing Science*.

[24] Laschinger H. K., Cummings G., Leiter M. (2016). Starting Out: a time-lagged study of new graduate nurses’ transition to practice. *International Journal of Nursing Studies*.

[25] Michie S., van Stralen M. M., West R. (2011). The behaviour change wheel: a new method for characterising and designing behaviour change interventions. *Implementation Science*.

[26] (1990). Statistical power analysis for the behavioral sciences. *Computers, Environment and Urban Systems*.

[27] Rong Y. F. (2009). The Relationship between Patient Safety Culture and Safety Behavior. https://xueshu.baidu.com/usercenter/paper/show?paperid=2b8ee4670797c559bc7be6957ed3e5ac&site=xueshu_se.

[28] Callister R. R., Kramer M. W., Turban D. B. (1999). Feedback seeking following career transitions. *Academy of Management Journal*.

[29] Kong Z. X., Li M. M. (2018). Research on creative feedback environments concept development, validation and comparison. *Science & Technology Progress and Policy*.

[30] Xue Y. R., Lin P., Gao X. Q. (2015). The development and reliability test of the new nurses’ transitional impact evaluation scale. *Chinese Journal of Nursing*.

[31] Klein R. B. (1998). Principles and Practice of Structural Equation Modeling. https://www.researchgate.net/publication/233896145_Principles_and_Practice_of_Structural_Equation_Modeling.

[32] Nunnally J. C. (1978). Psychometric theory. *American Educational Research Journal*.

[33] Wang A. M., Wang X. H., Du H. X., Yu C. L., Luan X. R. (2022). Path analysis of safety behavior status and related influencing factors of newly recruited nurses. *Chinese Journal of Nursing*.

[34] Chu X. Q., Li H. P., Ding X. T., Wu L. X., Zhang P. P. (2019). Studying on the relationship among nurses’ safe behaviors, head nurses’ diversified leading behaviors and patients’ safety culture perception. *The Chinese Health Service Management*.

[35] Liu Y., Teng W., Chen C., Zou G. (2022). Correlation of safety behavior, handover quality, and risk perception: a cross-sectional study among Chinese psychiatric nurses. *Frontiers in Psychiatry*.

[36] Kakemam E., Ghafari M., Rouzbahani M., Zahedi H., Roh Y. S. (2022). The association of professionalism and systems thinking on patient safety competency: a structural equation model. *Journal of Nursing Management*.

[37] Graf A. C., Jacob E., Twigg D., Nattabi B. (2020). Contemporary nursing graduates’ transition to practice: a critical review of transition models. *Journal of Clinical Nursing*.

[38] Wenxia Z., Feifei C., Min H., Li C., Aihong L., Xingfeng L. (2022). The status and associated factors of junior nurses’ transition shock: a cross-sectional study. *Journal of Nursing Management*.

[39] Labrague L. J., Santos J. A. A. (2020). Transition shock and newly graduated nurses’ job outcomes and select patient outcomes: a cross-sectional study. *Journal of Nursing Management*.

[40] Jørgensen J. T., Rozing M. P., Westendorp R. G. J. (2021). Shift work and incidence of psychiatric disorders: the Danish Nurse Cohort study. *Journal of Psychiatric Research*.

[41] Chin W., Guo Y. L., Hung Y. J., Yang C. Y., Shiao J. S. (2015). Short sleep duration is dose-dependently related to job strain and burnout in nurses: a cross sectional survey. *International Journal of Nursing Studies*.

[42] Zhu Y., Zhang Y., Wong F. K. Y. (2022). Newly graduated nurses’ stress, coping, professional identity and work locus of control: results of a cross-sectional study in Shanghai, Hong Kong and Taipei. *Journal of Nursing Management*.

[43] Murray M., Sundin D., Cope V. (2019). New graduate nurses’ understanding and attitudes about patient safety upon transition to practice. *Journal of Clinical Nursing*.

[44] Whitehead B., Owen P., Henshaw L., Beddingham E., Simmons M. (2016). Supporting newly qualified nurse transition: a case study in a UK hospital. *Nurse Education Today*.

[45] Buckner E. B., Anderson D. J., Garzon N., Hafsteinsdóttir T. B., Lai C. K., Roshan R. (2014). Perspectives on global nursing leadership: international experiences from the field. *International Nursing Review*.

[46] Pellico L. H., Maja D., Kovner C. T., Brewer C. S. (2010). Moving on, up, or out: changing work needs of new RNs at different stages of their beginning nursing practice. *Online Journal of Issues in Nursing*.

[47] Hampton K. B., Smeltzer S. C., Ross J. G. (2021). The transition from nursing student to practicing nurse: an integrative review of transition to practice programs. *Nurse Education in Practice*.

[48] Wiersma G., Pintz C., Fraser Wyche K. (2020). Transition to practice experiences of new graduate nurses from an accelerated bachelor of science in nursing program: implications for academic and clinical partners. *The Journal of Continuing Education in Nursing*.

[49] Hawkins N., Jeong S., Smith T. (2019). Coming ready or not! An integrative review examining new graduate nurses’ transition in acute care. *International Journal of Nursing Practice*.

[50] Vaismoradi M., Tella S., A Logan P., Khakurel J., Vizcaya-Moreno F. (2020). Nurses’ adherence to patient safety principles: a systematic review. *International Journal of Environmental Research and Public Health*.

[51] Crommelinck M., Anseel F. (2013). Understanding and encouraging feedback-seeking behaviour: a literature review: feedback-seeking behaviour: a review. *Medical Education*.

[52] Lee S. E., Quinn B. L. (2020). Safety culture and patient safety outcomes in east Asia: a literature review. *Western Journal of Nursing Research*.

[53] Alanazi F. K., Sim J., Lapkin S. (2022). Systematic review: nurses’ safety attitudes and their impact on patient outcomes in acute-care hospitals. *Nursing open*.

